# Theoretical and Experimental Analysis of Hydroxyl and Epoxy Group Effects on Graphene Oxide Properties

**DOI:** 10.3390/nano14080714

**Published:** 2024-04-19

**Authors:** Ximena Jaramillo-Fierro, Guisella Cuenca

**Affiliations:** 1 Departamento de Química, Facultad de Ciencias Exactas y Naturales, Universidad Técnica Particular de Loja, San Cayetano Alto, Loja 1101608, Ecuador; 2 Ingeniería Química, Facultad de Ciencias Exactas y Naturales, Universidad Técnica Particular de Loja, San Cayetano Alto, Loja 1101608, Ecuador; gpcuenca@utpl.edu.ec

**Keywords:** adsorbent materials, graphene oxide, methylene blue, density functional theory, water purification, environmental remediation

## Abstract

In this study, we analyzed the impact of hydroxyl and epoxy groups on the properties of graphene oxide (GO) for the adsorption of methylene blue (MB) dye from water, addressing the urgent need for effective water purification methods due to industrial pollution. Employing a dual approach, we integrated experimental techniques with theoretical modeling via density functional theory (DFT) to examine the atomic structure of GO and its adsorption capabilities. The methodology encompasses a series of experiments to evaluate the performance of GO in MB dye adsorption under different conditions, including differences in pH, dye concentration, reaction temperature, and contact time, providing a comprehensive view of its effectiveness. Theoretical DFT calculations provide insights into how hydroxyl and epoxy modifications alter the electronic properties of GO, improving adsorption efficiency. The results demonstrate a significant improvement in the dye adsorption capacity of GO, attributed to the interaction between the functional groups and MB molecules. This study not only confirms the potential of GO as a superior adsorbent for water treatment, but also contributes to the optimization of GO-based materials for environmental remediation, highlighting the synergy between experimental observations and theoretical predictions in advances in materials science to improve sustainability.

## 1. Introduction

Water pollution caused by industrial dyes represents a significant environmental issue [1]. During the dyeing process in the textile industry, between 10% and 25% of dyes are lost, and from 2% to 20% are directly discharged as aqueous effluents into the environment [2]. This results in the release of substantial quantities of dyes into water bodies, adversely affecting water quality, aquatic organisms, and human health. [3]. Beyond the visual impact, many dyes that are released into the water are toxic, carcinogenic, or mutagenic to living organisms, primarily due to the presence of aromatic compounds such as benzidine and naphthalene [4]. Methylene blue (MB), a widely used dye in the textile industry, has been classified as a high-risk compound due to its high toxicity and persistence in the environment [5].

For the removal of methylene blue and other dyes from water bodies, various physicochemical and biological methods have been developed [6,7] Adsorption, a physicochemical method, stands out for its low cost, simple operation and high efficiency, as well as the use of technological advancements to improve the process, including the development of new materials with a simple design [8,9,10]. This process relies on the electrostatic attraction between the contaminant molecules and the adsorption sites on the surface of the adsorbent [11], which occurs through various mechanisms, including electrostatic interactions, hydrogen bonding, and π–π interactions [12,13,14].

The search for effective adsorbents for dye removal, such as methylene blue from water, has led to the exploration of various materials, including graphene and its derivatives [15,16,17,18,19,20]. Graphene (G), composed of a single layer of carbon atoms arranged in a 2D hexagonal lattice [21,22], exhibits high electrical and thermal conductivity, granting it unique properties for contaminant adsorption [23,24]. Its high hydrophobic surface area and theoretical specific surface area make it an effective adsorbent [25]. Graphene oxide (GO), produced from graphene through oxidative processes [26,27,28,29,30], introduces functional groups that enhance adsorption capacity through chemical interactions [31,32,33,34,35]. Reduced graphene oxide (rGO) is derived from GO by reduction, partially restoring the graphene structure and its adsorption properties [36,37].

Recently, interest in the use of graphene-based materials such as methylene blue for water decontamination has increased, leveraging their large surface area and porous structure for efficient dye molecule adsorption, with removal efficiencies exceeding 95% [38,39,40]. Experimental and computational studies, including density functional theory (DFT) analyses, have been conducted to elucidate the adsorption capacity of MB on graphene, GO, and rGO, providing insights into the molecular adsorption mechanisms and predicting material adsorption capacities [41,42,43,44,45,46].

Over the last century, the atomic structure of graphene oxide has been investigated, with various models proposed for its functional group composition and arrangement. Experimental confirmations have identified epoxy and hydroxyl groups, and sp^2^ coordinated carbons in carbon layers. Likewise, previous studies have identified carbonyl and carboxyl functional groups at the edges of carbon layers. These experimental findings also suggest the presence of non-oxidized aromatic “islands”, separated by aliphatic rings with hydroxyl groups, epoxy, and double bonds. Furthermore, theoretical research highlights the role of epoxy and hydroxyl groups in structural stability, suggesting a model where carbon layers exhibit bulges and a random distribution of oxygen-containing groups [47,48].

Despite numerous models, the precise structure of graphene oxide remains unresolved. Therefore, this study aims to understand the atomic structure of graphene oxide due to its significant impact on electronic properties and, consequently, on methylene blue adsorption capacity. The experimental and theoretical results presented here shed light on the molecular adsorption of MB dye by graphene oxide. Furthermore, DFT calculations of pristine graphene (G), hydroxyl-decorated graphene (GOL), epoxy-decorated graphene (GOXI), and graphene decorated with both functional groups (GOL/GOXI) are explored to determine their influence on the electronic properties of the material. Using the Vienna Ab Initio Simulation Package (VASP), the molecular interactions and adsorption mechanisms on G, GOL, and GOXI surfaces are investigated, comparing the effects of hydroxyl and epoxy groups on MB adsorption capacity. Additionally, the experimental results aim to establish the influence of solution pH, dye concentration, reaction temperature, and contact time on graphene oxide’s ability to adsorb methylene blue dye.

The novelty of this study lies in its comprehensive approach that combines detailed experimentation and theoretical analysis to clarify the adsorption mechanism of methylene blue (MB) on graphene oxide (GO). This research highlights, for the first time, the synergisms between experimentation and theory to achieve an in-depth and detailed understanding of the molecular-level interactions between GO and MB. By exploring the effect of critical operational variables and detailing the vital role of functional groups in enhancing adsorption, this study not only advances the scientific knowledge of adsorbent materials but also opens new perspectives aimed at the development of more efficient and sustainable technologies for wastewater treatment.

## 2. Materials and Methods

### 2.1. Materials

All chemicals were used as received, without further purification: graphite powder (<150 µm, 99.99%, Sigma-Aldrich, Burlington, MA, USA); hydrochloric acid (HCl, 37%, Sigma-Aldrich, Burlington, MA, USA); hydrogen peroxide (H_2_O_2_, 30%, Merk, Darmstadt, Germany); methylene blue (MB, C_16_H_18_N_3_ClS, Sigma-Aldrich, Burlington, MA, USA); potassium permanganate (KMnO_4_, ≥99.0%, Sigma-Aldrich, Burlington, MA, USA); sodium hydroxide (NaOH, 1310-73-2, 40.00 g/mol, Merk, Darmstadt, Germany); sulfuric acid (H_2_SO_4_, 95.0–98.0%, Sigma-Aldrich, Burlington, MA, USA), Methanol (CH_3_OH, ≥99.8%, Sigma-Aldrich, Burlington, MA, USA).

### 2.2. Graphene Oxide (GO) Synthesis

GO was obtained using the modified Hummers method [49]. A total of 3.0 g of powdered graphite was added to 70 mL of sulfuric acid (H_2_SO_4_) with constant stirring. Then, 9.0 g of potassium permanganate (KMnO_4_) was added with moderate stirring and placed in an ice bath, where it was kept for 30 min. Next, the solution was transferred to a water bath at a temperature of 50 °C with constant stirring for 30 min. Then, 150 mL of distilled water (H_2_O) was added and stirred for 20 min, maintaining a temperature below 90 °C. A total of 500 mL of H_2_O and 15 mL of 30% hydrogen peroxide (H_2_O_2_) were added. The resulting solution was kept at rest for 24 h at room temperature. Subsequently, the precipitate was centrifuged at 1000 rpm for 12 min and washed with 15 mL of hydrochloric acid (HCl) (1:10); this was carried out three times. Again, it was centrifuged at 1000 rpm for 12 min and washed with distilled H_2_O; this continued until a pH of around 7.00 was reached. Finally, the sample was transferred to a beaker and dried at 100 °C in the oven for 24 h. After the oxidation process, 100 mg of the sample was dispersed in 1 L of distilled water via sonication for 30 min. The resulting solution was centrifuged to separate graphene oxide (GO) from non-exfoliated graphite oxide particles.

### 2.3. Characterization of Graphene Oxide (GO)

The characterization of samples was completed through applying the methodology described in our previous study [50]. X-ray diffraction (XRD) measurements were made using a Bruker-AXS D8-Discover diffractometer (Bruker AXS, Karlsruhe, Germany) equipped with Cu Kα radiation (λ = 1.5406 Å). The identification of crystalline phases was conducted via the Crystallography Open Database (COD, version 2023). For microstructural analysis, micrographs and energy-dispersive X-ray (EDX) spectra were acquired with a JEOL JSM 6400 scanning electron microscope (SEM) (JEOL, Peabody, MA, USA), which was fitted with an in-house-developed JEOL dispersive X-ray spectrometer (EDS). Fourier-transform infrared (FTIR) spectra were captured on a PerkinElmer GX2000-FTIR Spectrometer (PerkinElmer, Inc., Waltham, MA, USA). The specific surface area (SSA) of graphene oxide (GO) was determined by liquid nitrogen physisorption at −196 °C using a ChemiSorb 2720 instrument (Micromeritics, Norcross, GA, USA). The gas mixture used for the analysis consisted of 30% nitrogen (N_2_) in helium (He). The SSA calculation was based on the Brunauer–Emmet–Teller (BET) equation, utilizing the Chemisoft TPx system (version 1.03; Micromeritics, 2011) for data analysis via the single-point method.

The point of zero charge (PZC) of GO was assessed at room temperature (20 ± 2 °C). The methodology involved adding 0.1 g of GO powder to a 50 mL tube containing 25 mL of a 0.1 M NaCl solution. The pH was adjusted to values between 3 and 10 using 0.1 M HCl or NaOH, recorded as pH_i_. Following 24 h of agitation at 250 rpm, the pH of the supernatant (pH_f_) was measured. The pH_PZC_ was determined at the intersection of the experimental curve (initial pH versus final pH) with the line defined by x = y (initial pH = final pH). This procedure was replicated three times to calculate the average pH_PZC_ value for GO [51,52]. Lastly, the quantification of residual methylene blue (MB) in solutions was performed using a Jenway 7350 spectrophotometer (Cole-Parmer, Staffordshire, UK) at a wavelength of 623 nm.

### 2.4. Experimental Methodology

This investigation conducted a series of adsorption experiments utilizing graphene oxide (GO) with aqueous solutions of methylene blue (MB) to evaluate the effects of various parameters, including solution pH, initial adsorbate concentration, reaction system temperature, and adsorbate–adsorbent contact time. The acquired data were analyzed through fitting with isotherm and kinetic models using the least-squares non-linear regression method [53].

The adsorption capacity of GO (100 mg L^−1^) for an MB solution (10 mg L^−1^) was assessed across a pH range from 3 to 12, adjusting the pH with 0.1 M HCl and 0.1 M NaOH solutions. These solutions were stirred continuously for 24 h at ambient temperature before measuring the residual MB concentration. Further adsorption experiments were performed in a batch reactor at a controlled pH of 7.0 ± 0.1 and at room temperature (20 ± 2.0 °C), using 200 mg L^−1^ of GO. To explore the maximum adsorption capacity for MB, the concentration of a 500 mL MB solution was varied from 2.5 to 50 mg L^−1^. Adsorption thermodynamics and kinetics for MB were investigated using a 20 mg L^−1^ MB solution in a 500 mL volume, with absorbance measurements at 623 nm taken using a UV-visible spectrophotometer and a pre-established calibration curve (R^2^ = 0.9987) according to the Lambert–Beer Law. Experiments were performed in triplicate, and results were reported as mean values. The amount of dye adsorbed onto GO was calculated using Equation (2) [54], as presented in Table 1.

Equilibrium adsorption of MB was analyzed using the Langmuir [55], Freundlich [56], and Temkin [57] isotherm models, represented by Equations (3)–(5), respectively, as detailed in Table 1. Additionally, the adsorption heat (B) and separation factor (RL) constants were calculated using Equations (6) and (7) [55], providing insight into the adsorption characteristics.

Thermodynamic parameters, including Gibbs free energy (∆G^0^), enthalpy (∆H^0^), and entropy (∆S^0^), were determined using Equation (8) in Table 1 [58]. The relationship between these parameters was established using the van’t Hoff equation, represented by Equation (9) in Table 1. The dimensionless parameter k_C_ was calculated by multiplying k_L_ by the molecular weight of the adsorbate (M_w_) and adjusting for the presence of 1000 moles of pure water per liter, as detailed in Equation (10) [59] in Table 1.

Adsorption kinetics were examined using pseudo-first-order, pseudo-second-order, Elovich, intraparticle diffusion, external-film diffusion, and internal-pore diffusion models [56,57], each described by mathematical expressions (11) through (16), as outlined in Table 1. Finally, GO was desorbed after one treatment cycle to evaluate its recyclability. MB desorption from the loaded adsorbent was achieved using pure methanol. The adsorbent was then dried and reused under identical conditions for subsequent cycles. This recycling process was repeated over three cycles [60]. In each cycle, a new 500 mL MB solution (20 mg L^−1^) was used with the GO concentration maintained at 200 mg L^−1^.

### 2.5. Theoretical Methodology

The Density Functional Theory (DFT) investigation was conducted following the methodology outlined in our previous study [50]. Theoretical calculations were carried out using the Vienna Ab Initio Simulation Package (VASP) version 6.0, developed by VASP Software GmbH in Vienna, Austria [61,62]. Molecular modeling and visualization were performed using BioVia Materials Studio, version 5.5, provided by BioVia in San Diego, CA, USA.

The ionic potential of both inner nuclei and electrons was represented using pseudopotentials, which utilized the projector augmented wave (PAW) methodology [63]. All calculations employed the Perdew–Burke–Ernzerhof (PBE) generalized gradient approximation (GGA) function to describe electronic exchange–correlation interactions, which is known for its effectiveness in characterizing interactions between adsorbates and various surfaces, including sp^2^ carbon-based materials [64]. The plane wave cutoff energy was established at 500 eV. Kohn–Sham equations were solved in a self-consistent manner, ensuring that energy variations between computational cycles remained below 10^−5^ eV [65].

Recognizing the limitation of GGA functionals, including hybrids, in describing the long-range electron correlations crucial for van der Waals (vdW) forces, this study incorporated the Grimme dispersion correction (DFT-D2) approach. This was particularly relevant for accurately modeling interactions in systems involving large atoms/molecules, such as those with aromatic rings [66,67,68]. The dispersion correction was applied to simulate both the surface structure and the adsorption of MB molecules onto the GO surface.

Given the complex nature of the structure and chemical composition of GO, it is generally accepted that epoxy (–C–O–C–) and hydroxyl (–OH) functional groups are randomly distributed on the GO sheet surface, while carboxyl (–COOH) and carbonyl (–C=O) groups are predominantly found at the edges [69]. The theoretical adsorption study thus considered two configurations of functionalized graphene oxide, focusing on models with either epoxy or hydroxyl groups.

The simulation of methylene blue (MB) molecule adsorption onto GO surfaces utilized optimized lattice parameters: a = 22.22 Å, b = 38.48 Å, and c = 16.00 Å, with the angles between the lattice vectors set to <90° × 90° × 120°> [70]. The dynamic configuration adopted point symmetry P1, and the Brillouin zone was sampled using Γ-centered (2 × 1 × 2) Monkhorst–Pack grids [71]. Simulations were performed under non-spin-polarized conditions, with atomic positions relaxed until forces on the atoms were reduced to below 0.01 eV/Å. The GO model was represented by a p(5 × 5) supercell containing 370 carbon atoms, two oxygen-containing groups, and a 15 Å vacuum region in the out-of-plane direction. Surface energies (γs) of the GO structures were calculated following Equation (17), as outlined in Table 1 [72,73]. The adsorption energy (E_ads_) of MB molecules on the surfaces of graphene oxide was determined using Equation (18) in Table 1 [74,75].

For a deeper understanding of the chemical interactions between MB and GO, a population analysis was conducted using Bader’s method, a valuable approach for assessing bond ionicity by quantifying the charge transfer between atoms [76,77]. Charge difference analysis further quantified the redistribution of charge on the GO surface due to MB adsorption [76]. Additionally, the electron localization function (ELF) was employed to gain insights into the nature of MB-GO interactions [78], where the distribution of maximum electron density (Region of Maximum Density (RMD)) around atomic nuclei provides information on the interaction type. Symmetric RMD distribution suggests ionic or van der Waals interactions, while the migration of RMD between atomic centers indicates increasing covalent character, achieving ideal covalent symmetry [79].

## 3. Results

### 3.1. Characterization of GO

#### 3.1.1. XRD Analysis

**Figure 1:**
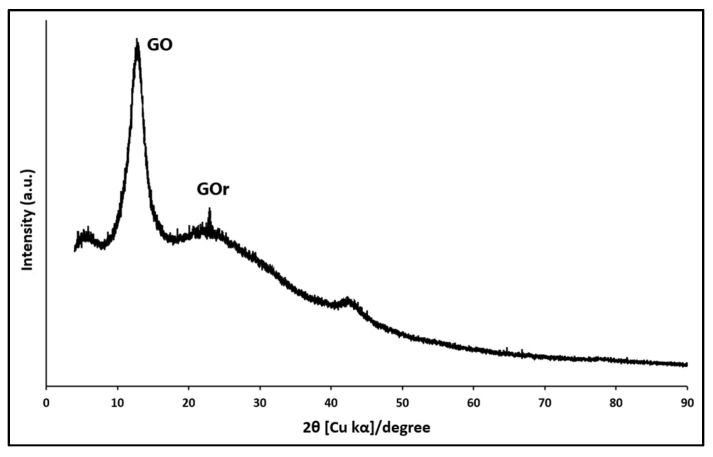
X-ray diffraction pattern of GO synthesized using the Hummers method.

Figure 1 presents the X-ray diffraction pattern obtained for the synthesized material. According to the literature, graphite shows a sharp peak at approximately 2ϴ = 26.00° (d = 0.34 nm), which corresponds to the diffraction peak (0 0 2), while, due to the increase in the distance between layers, graphite oxide graphite and graphene show a sharp peak at approximately 2ϴ = 11.15° (d = 0.81 nm) that corresponds to the diffraction peak (0 0 1) [80,81]. As can be seen in Figure 1, the diffraction pattern agrees with the literature, since it shows a peak at 2ϴ = 12.12° (d = 0.73 nm) that corresponds to the (0 0 1) diffraction peak of graphene oxide. Furthermore, another peak is observed at 2ϴ = 22.87°, characteristic of reduced graphene oxide, indicating that part of the graphene oxide was reduced during the synthesis process.

#### 3.1.2. SEM and EDS Analysis

**Figure 2:**
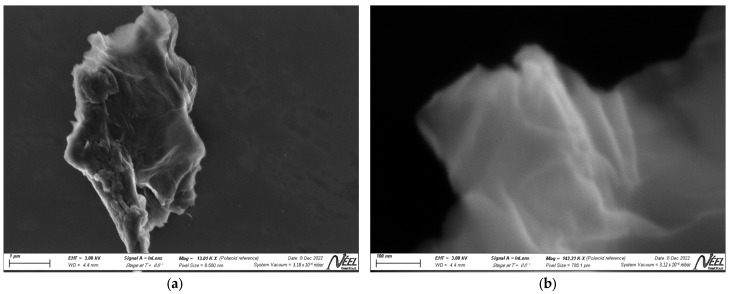
SEM micrographs of GO at magnifications of (**a**) 1 μm and (**b**) 100 nm.

To study the morphology of the synthesized material surface, an SEM analysis was performed. Figure 2 shows a somewhat rough structure of the material, with sheets folded over each other. The GO morphology shown at different magnifications in Figure 2a,b indicates that the exfoliation process was carried out successfully and some GO layers were synthesized.

**Figure 3:**
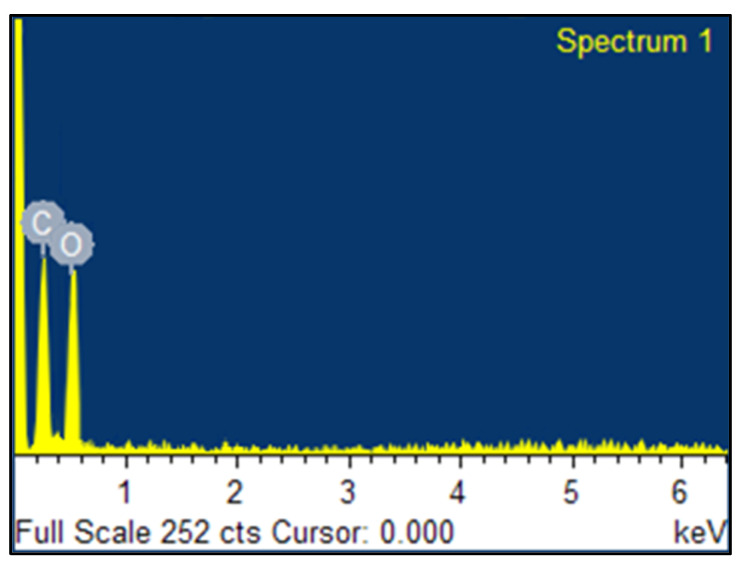
EDS spectra of synthesized GO.

To investigate the composition of the synthesized material, EDS measurements were performed. The results shown in Figure 3 reveal that there was only elements C (52.6%) and O (47.4%) were present in the synthesized material, suggesting high purity or no source of significant contamination for the obtained product.

#### 3.1.3. FTIR Analysis

**Figure 4:**
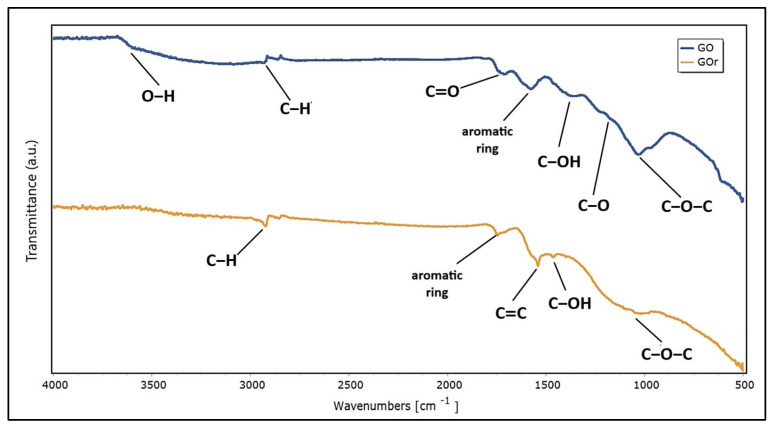
Fourier transform infrared spectra of GO and GOr.

The presence of oxygen functional groups, especially hydroxyl, epoxy and carboxyl, which are key to the functionality of GO [82], was confirmed by FTIR analysis. The existence of these groups demonstrates the good oxidation of graphite. Figure 4 shows the spectrum of GO and GOr to compare and analyze their structural and functional differences.

**Figure 5:**
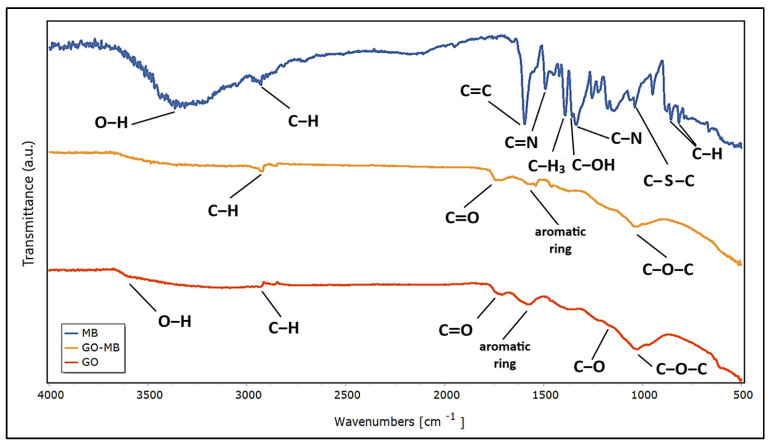
Fourier transform infrared spectra of MB, GO-MB and GO.

Likewise, the adsorption of MB dye on GO was confirmed by FTIR analysis. Figure 5 shows the spectrum of MB, GO-MB, and GO, illustrating the changes in functional groups and molecular interactions upon dye adsorption.

### 3.2. Experimental Adsorption Studies

#### 3.2.1. Effect of pH on MB Adsorption

**Figure 6:**
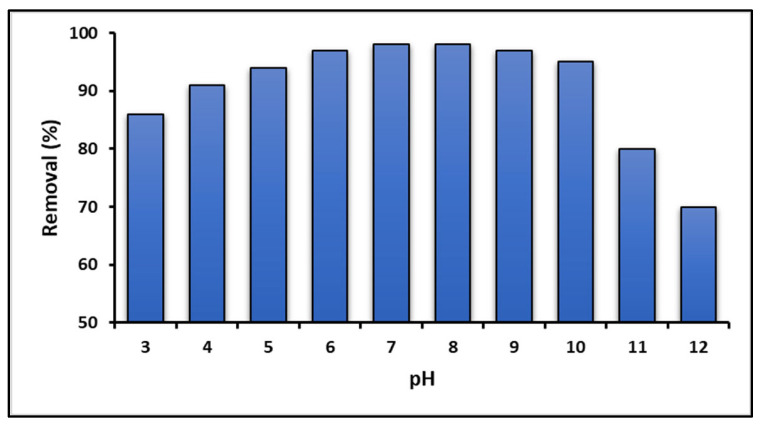
Effect of solution pH on MB adsorption (GO concentration = 100 mg L^−1^; MB concentration = 10 mg L^−1^; temperature = 20 ± 2.0 °C; time = 24 h).

Figure 6 illustrates the ability of graphene oxide (GO) to remove MB dye as a function of the solution pH. This figure reveals that the dye can be adsorbed at acidic pHs below PZC (pH_PZC_ = 5.5). As the pH of the dye solution increases, the adsorption of cationic MB dye molecules on the GO surface is enhanced. However, Figure 6 also shows a decrease in adsorption capacity starting at pH > 10, which could be due to the hydrolysis of GO [83]. According to the literature, GO is stable at pH values between 4 and 10 [84].

#### 3.2.2. Effect of Initial Concentration of MB

**Figure 7:**
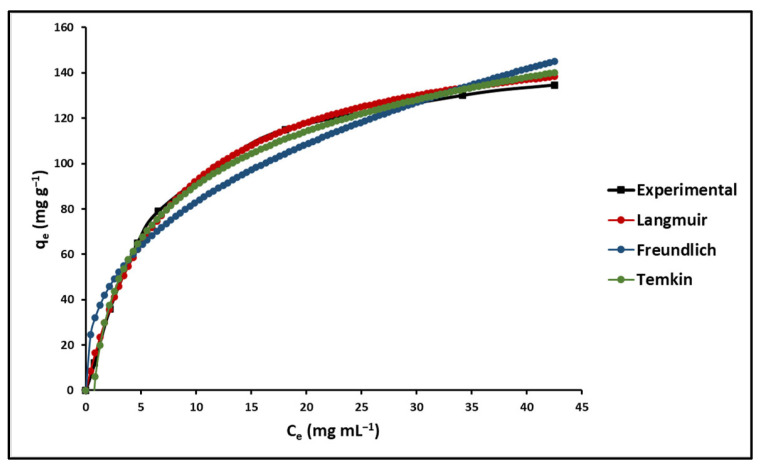
Effect of concentration on MB adsorption (GO concentration = 200 mg L^−1^; solution pH = 7.0 ± 0.1; temperature = 20 ± 2.0 °C).

The effect of the initial concentration of the dye is closely related to its concentration and the sites present on the adsorbent surface [85]. The effect of the initial concentration of MB on the adsorption capacity of GO was determined by measuring the amount of residual MB in the solution via UV-Vis spectrophotometry at λ = 623 nm and applying Equation (1), as shown in Table 1. The adsorption maximum of GO was calculated using equilibrium isotherms at different initial concentrations of MB, from 1.5 to 50 mg L^−1^. The fit of the experimental data is presented in Figure 7 for the Langmuir, Freundlich and Temkin isotherm models, according to Equations (2)–(4), as shown in Table 1, respectively. According to Figure 7, a better fit was demonstrated with the Langmuir isotherm model.

The parameters calculated from the isotherms are presented in Table 2. The estimated constants for the Langmuir, Freundlich, and Temkin models at absolute temperatures of 293.15 K, 303.15 K, and 313.15 K are also displayed in Table 2. In this table, the values of the separation factor or equilibrium parameter (R_L_) are in the range of 0–1. Similarly, the coefficient “n”, which reports the adsorption intensity, is within the range of 1–10.

#### 3.2.3. Effect of Temperature on MB Adsorption

**Figure 8:**
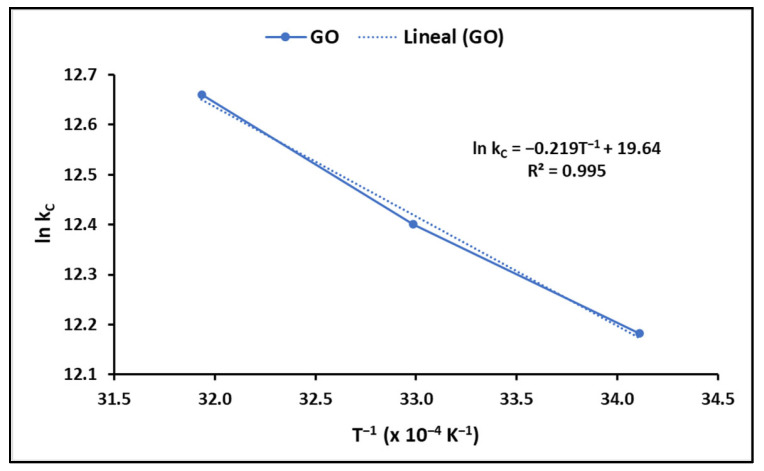
Temperature effect on MB adsorption (GO concentration = 200 mg L^−1^; solution pH = 7.0 ± 0.1; temperature = 20 ± 2.0 °C).

It is widely known that the adsorption process depends on the temperature of the solution. In this context, thermodynamic parameters provide insight into the viability and spontaneity of a process [86]. To determine these thermodynamic parameters, in particular the Gibbs free energy change (∆G°), enthalpy change (∆H°), and surface entropy change (∆S°), the equilibrium constant was established at various temperatures, according to Equations (7)–(9), as shown in Table 1. Figure 8 displays the results of this analysis.

The values of the thermodynamic parameters obtained in this study are described in Table 3. ∆G° indicates the degree of spontaneity in the process, and the more negative the values are, the more favorable the adsorption. On the other hand, positive values of ΔH° suggest that the process is endothermic, while positive values of ΔS° suggest an increase in randomness at the solution–solid interface during the adsorption process.

#### 3.2.4. Effect of Contact Time on MB Adsorption

**Figure 9:**
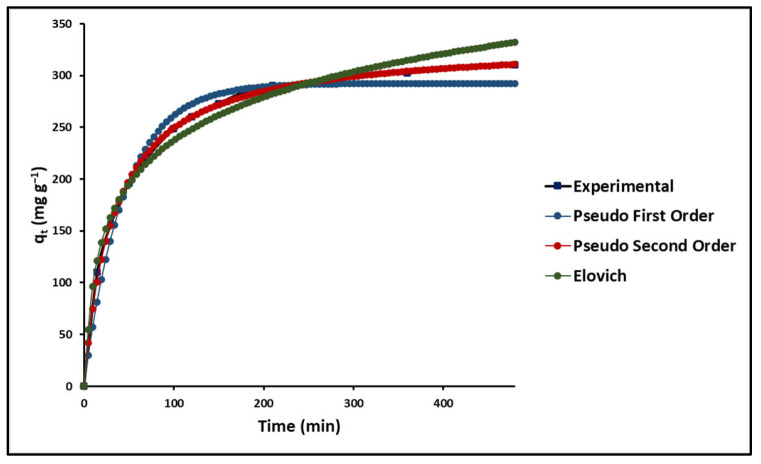
Time effect on MB adsorption (GO concentration = 200 mg L^−1^; MB concentration = 20 mg L^−1^; solution pH = 7.0 ± 0.1; temperature = 20 ± 2.0 °C).

In this study, the kinetics of the adsorption process of MB on GO were investigated. For this, three models were used: the pseudo-first-order model (Lagergren), the pseudo-second-order model (Ho), and the Elovich Model. Figure 9 shows the fit of the experimental data based on Equations (10)–(12), as presented in Table 1.

As shown in Figure 8, all models exhibit an initial phase of rapid adsorption followed by a second phase of slow adsorption. Table 4 shows that the pseudo-second-order model best describes the kinetic behavior of the dye adsorption process on the GO surface, which agrees with the reports of other authors [87].

**Figure 10:**
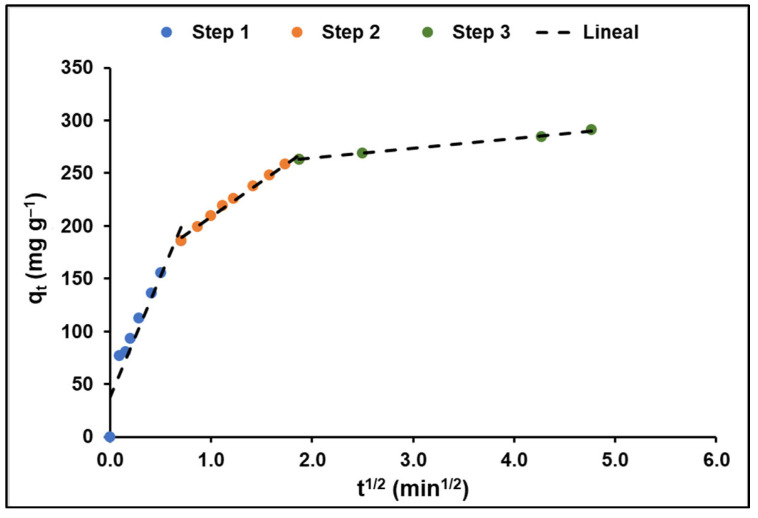
Diffusional effects on MB adsorption (GO concentration = 200 mg L^−1^; MB concentration = 20 mg L^−1^; solution pH = 7.0 ± 0.1; temperature = 20 ± 2.0 °C).

On the other hand, the adjustment of the intraparticle diffusion model (Figure 10) shows, according to Equation 13 of Table 1, that the adsorption of MB on GO takes place in three stages: an initial stage of fast speed (k_1_ = 231.45 mg g^−1^ min^−1/2^), another, slower, intermediate stage (k_2_ = 66.85 mg g^−1^ min^−1/2^), and a final stage that tends to equilibrium (k_3_ = 9.28 mg g^−1^ min^−1/2^). The values of the kinetic constants for each stage, as well as the values of the effective diffusion coefficients D_f_ and D_p_, calculated using Equations (14) and (15) of Table 1, respectively, are shown in Table 4.

From Figure 10, it is evident that in the first, higher-speed, stage, MB molecules diffuse from the liquid film toward the adsorbent surface. In the second stage, the diffusion rate was lower, probably because the adsorption process occurred. In the last phase, the equilibrium trend was characterized by a much lower rate due to the decrease in MB concentration in the aqueous solution and the reduction in active sites in the adsorbent.

#### 3.2.5. Desorption and Reusability of GO for MB Removal

**Figure 11:**
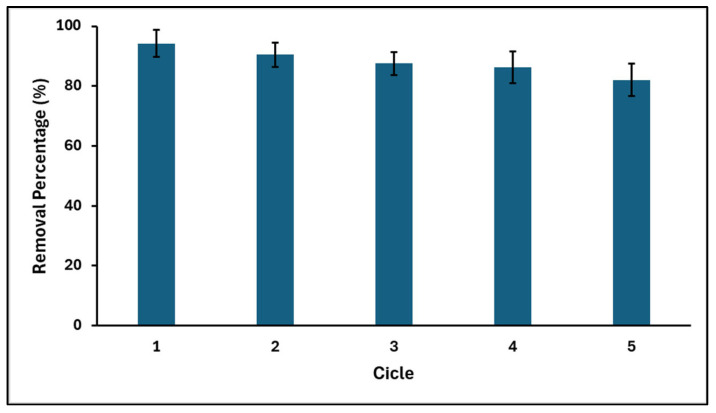
Recovery efficiency of GO after MB adsorption (GO concentration = 200 mg L^−1^; MB concentration = 20 mg L^−1^; solution pH = 7.0 ± 0.1; temperature = 20 ± 2.0 °C).

Since the recycling and regeneration of the adsorbent is very important in practical applications, GO recycling experiments were carried out for five consecutive cycles. After each cycle, the materials were washed with methanol to desorb the adsorbed dye and were again subjected to the adsorption process, maintaining the same operational conditions as the previous cycle. Figure 11 shows that the recovery efficiency of GO is generally high, and the adsorption capacity is little affected over five consecutive adsorption–desorption cycles. In fact, after the fifth cycle, the MB adsorption capacity of GO decreased by only 12%. From these results showing a high recycling efficiency, it is suggested that the GO synthesized in this study is suitable for practical application.

### 3.3. Computational Studies

#### 3.3.1. Optimization of Adsorbent Structures

In this study, the effect of the hydroxyl and epoxy functional groups in graphene was determined using 3 × 3 supercells. These groups are common in oxidized graphene and are considered impurities in its structure. Several experimental studies have shown that oxidized graphene sheets have a thickness that is an integer multiple of approximately 6.7 Å, suggesting the likely presence of hydroxyl and epoxy groups on both sides of the graphene sheet [69]. However, the focus of this work is on possible configurations on a single side of the graphene oxide sheet.

**Figure 12:**
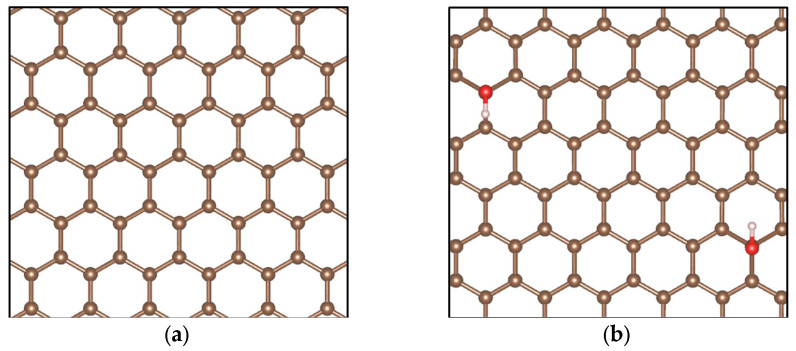

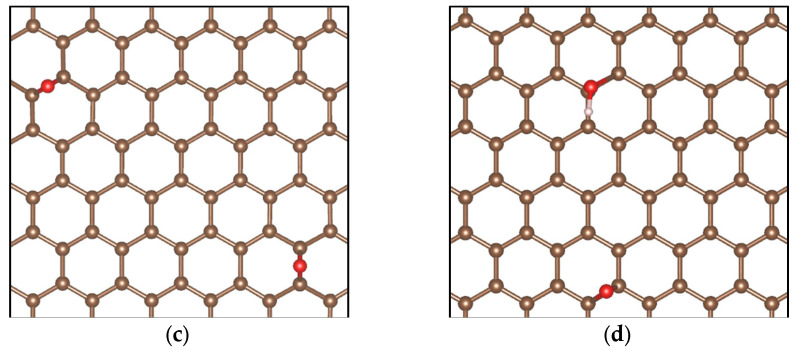
Simulation models of (**a**) pristine graphene (G), (**b**) GO-hydroxyl (GOL), (**c**) GO-epoxy (GOXI), and (**d**) GO-hydroxyl/epoxy (GOL/GOXI). The brown balls are C atoms, the red balls are O atoms, and the white balls are H atoms.

Figure 12 shows the simulation models for pristine graphene (G), graphene oxide decorated with only one hydroxyl group (GOL), graphene oxide decorated with only one epoxy group (GOXI), and graphene oxide decorated with both hydroxyl and epoxy groups (GOL/GOXI). It is worth mentioning that analyzing the hydroxyl and epoxy functional groups, in isolation and in combination, is essential to understand the effects of impurity dispersion and doping in graphene [88].

As can be seen in Figure 12, the hydroxyl group forms a bond perpendicular to the plane of graphene, causing changes in bond lengths and angles. On the other hand, the epoxy group establishes a bond with two adjacent carbon atoms, which generates distortions in the graphene structure. The structures obtained in our calculations agree well with those reported in other theoretical studies [47,48].

#### 3.3.2. Electronic Properties of Adsorbent Structures

**Figure 13:**
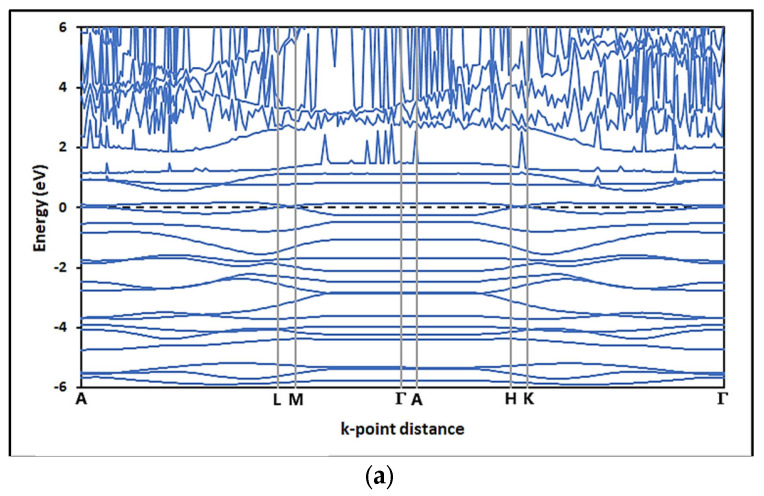

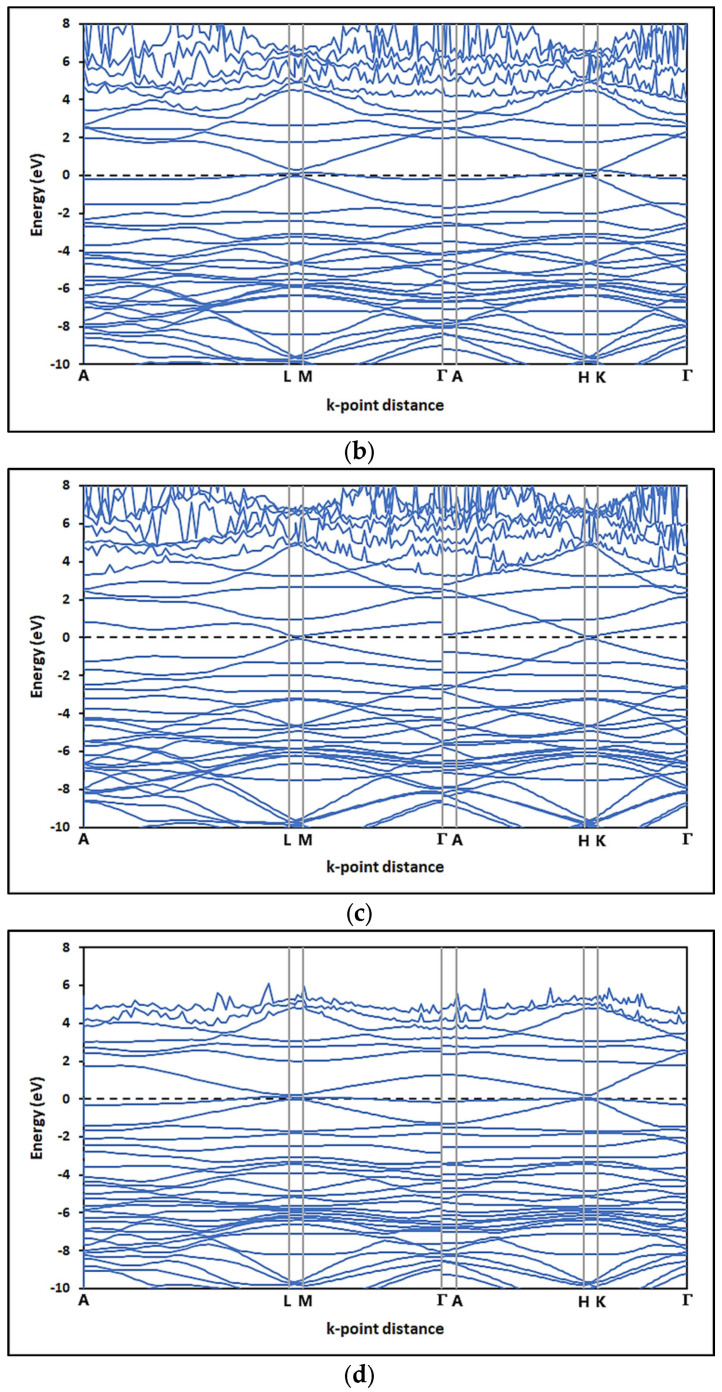
Band structure (BS) of (**a**) pristine graphene (G), (**b**) GO-hydroxyl (GOL), (**c**) GO-epoxy (GOXI), and (**d**) GO-hydroxyl/epoxy (GOL/GOXI).

In this study, the electronic properties of pristine graphene and graphene oxides were determined by analyzing the band structure (SB) and the density of states (DOS). Figure 13 shows the band structures (SB) for the configurations of (a) pristine graphene (G) and the graphene oxides—(b) GOL, (c) GOXI, and (d) GOL/GOXI—along the high-symmetry directions A—L—M—Γ—A—H—K [89]. This figure shows that the presence of oxygenated functional groups in the graphene monolayers does not shift the Dirac point regarding the Fermi level.

**Figure 14:**
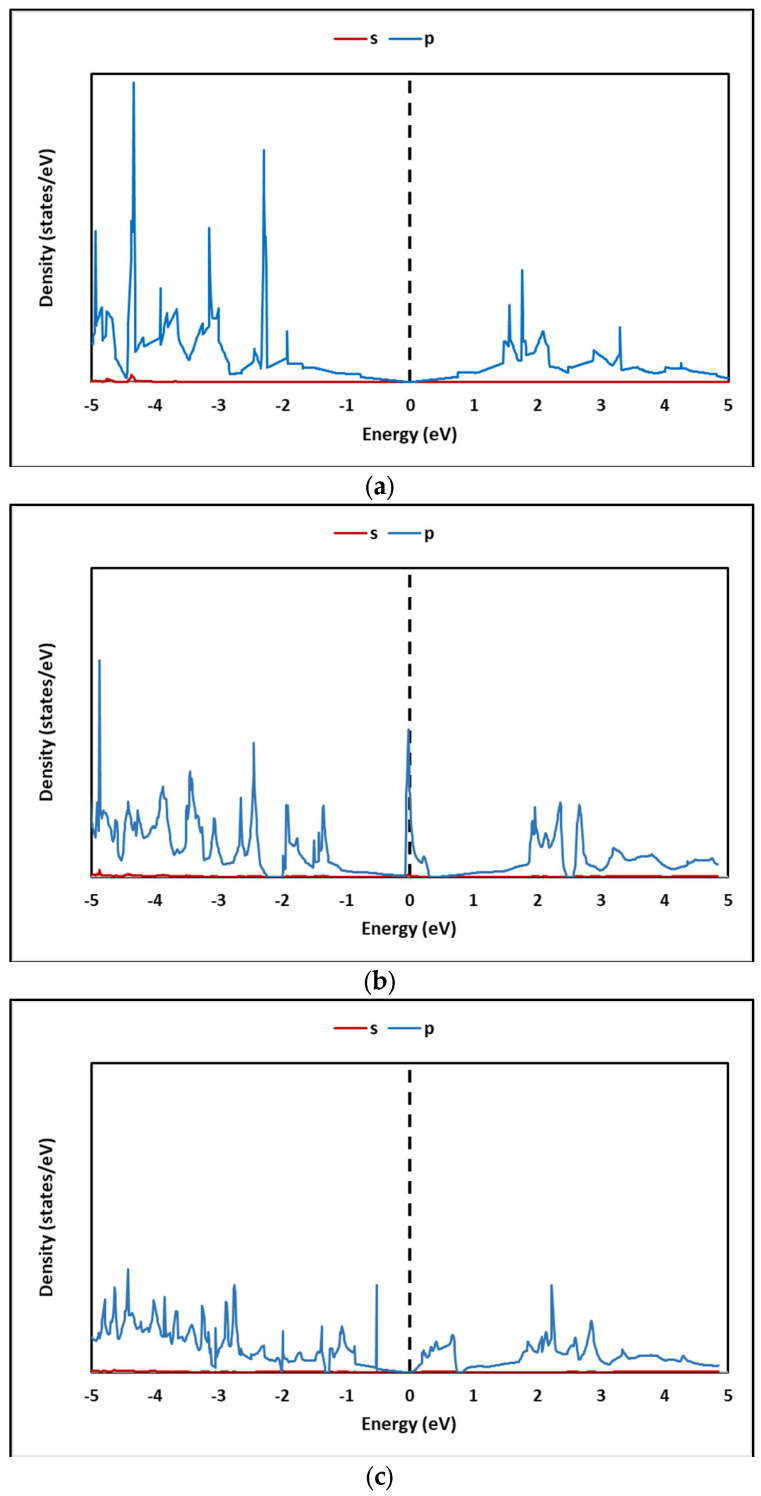

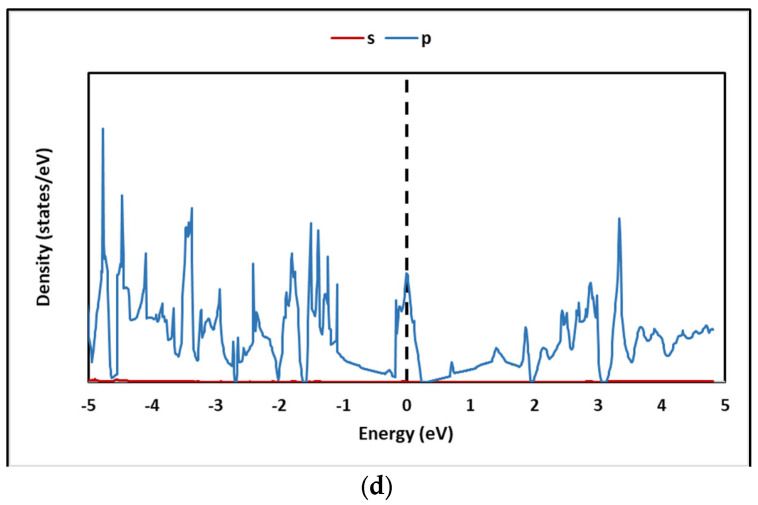
Density of states (DOS) of (**a**) pristine graphene (G), (**b**) GO-hydroxyl (GOL), (**c**) GO-epoxy (GOXI), and y (**d**) GO-hydroxyl/epoxy (GOL/GOXI).

On the other hand, the DOS of pristine graphene (G) and graphene oxides GOL, GOXI, and GOL/GOXI are shown in Figure 14a–d, respectively. Figure 14b,c show that the GOL and GOL/GOXI oxides present a sharp peak in the density of states near the Fermi level, which indicates the presence of highly localized electronic states in this region. Furthermore, the comparison between the density of states of pristine graphene and graphene oxides highlights the differences in the electronic properties of these materials, which has important implications for the design and development of electronic devices and advanced materials.

Figure 14a shows the density of states (DOS) of pristine graphene (G), evidencing the location of the Fermi level at zero. The DOS of Figure 14b shows, for the GOL oxide, the existence of a distinctive peak at the Fermi level, which could be induced by the neutral OH group with an odd number of electrons in the supercell. In Figure 14c, the epoxy group of the GOXI oxide eliminates the localized state at the Fermi level, generating a small band gap (approximately 0.1 eV) in the DOS of the system. Finally, in Figure 14d, the DOS of GOL/GOXI oxide shows a similar trend to GOL oxide, with a distinct peak at the Fermi level.

#### 3.3.3. Computational Adsorption Studies

**Figure 15:**
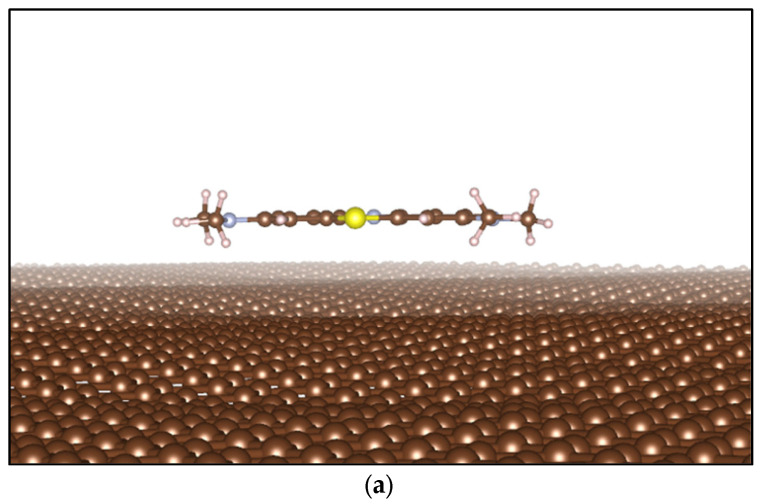

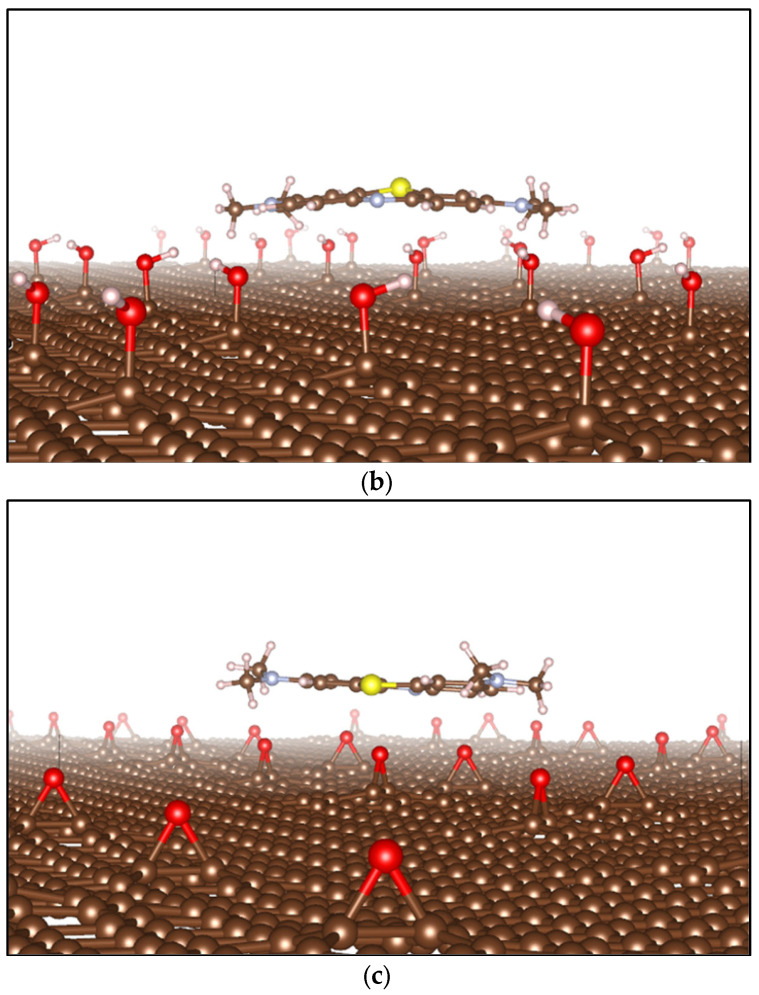
Optimized structures of (**a**) MB adsorbed on Graphene (G), (**b**) MB adsorbed on GOL oxide, and (**c**) MB adsorbed on GOXI oxide. The brown balls are C atoms, the red balls are O atoms, the white balls are H atoms, the gray balls are N atoms, and the yellow balls are S atoms.

Figure 15 shows the simulation models for MB adsorption on the surfaces of pristine G (graphene), GOL (graphene oxide decorated with a hydroxyl group), and GOXI (graphene oxide decorated with an epoxy group). This figure shows that the hydroxyl and epoxy functional groups are randomly distributed in the respective GO monolayers. As can be seen from Figure 15b,c, the presence of hydroxyl and epoxy groups can cause significant local distortion in the structure of pristine graphene.

Table 5 summarizes the values of adsorption energy, interfacial distance, and transferred charge for the optimized reaction systems. By convention, transferred load values show a (^+^) sign when the structure gains load (e) and a (^−^) sign when it loses load (e).

The most energetically stable adsorption for the MB molecule occurred on graphene oxide (GO) structures, and was higher for GOL than for GOXI. These results were confirmed in the Bader analysis, which demonstrated a greater charge transfer for the GOL-MB system, as seen in Table 5.

Likewise, to verify the chemical interaction between the adsorbent surfaces (G, GOL, and GOXI) and the MB molecule, a population analysis of the G-MB, GOL-MB, and GOXI-MB systems was performed using the Bader method [77,90]. This analysis is valuable as it allows for a description of the ionicity of chemical bonds by evaluating the charge transfer between the bonded atoms [76]. Table 6 shows, for each system, the average charge per atom type, before (BA) and after (AA) MB adsorption. The atoms belonging to the adsorbent surface were designated with the subscript (_surf_) while those belonging to the methylene blue molecule were designated with the subscript (_MB_).

**Figure 16:**
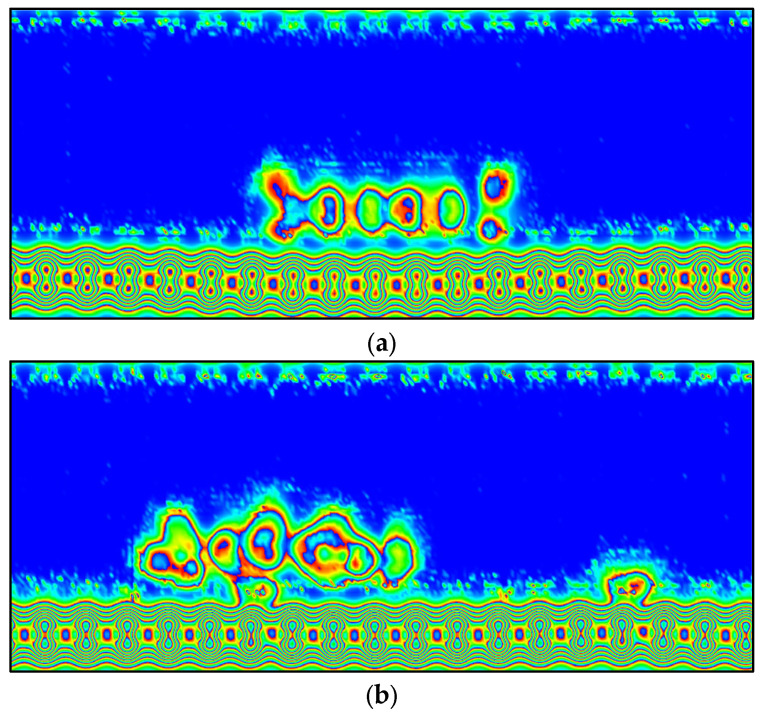

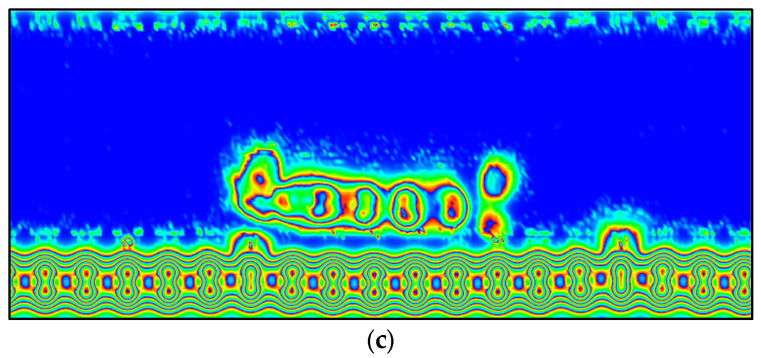
ELF analysis of (**a**) MB adsorbed on graphene (G), (**b**) MB adsorbed on GOL graphene oxide (GOL), and (**c**) MB adsorbed on GOL graphene oxide (GOXI). The red, green, and blue (RGB) color scale for ELF analysis represents electronic localization (dark red) to electronic delocalization (light blue).

Furthermore, the electron localization function (ELF) was used in this study to gain a deeper understanding of the G-MB, GOL-MB, and GOXI-MB interactions [78]. In the ELF analysis, when the region of maximum density (RMD) is more symmetrically distributed around the nucleus, it means that a more ionic or Van der Waals interaction is taking place. In contrast, as the covalent character of a bond strengthens, RMD migration between centers becomes more pronounced until a perfectly symmetric geometry is achieved in the ideal covalent scenario. The ELF section for the G-MB, GOL-MB, and GOXI-MB interactions is shown in Figure 16. As can be seen in this figure, there is a slightly stronger interaction between the MB molecule and the hydroxyl functional group of graphene oxide. The figure shows that the RMDs are located along the line connecting the nuclei and can be separated from the nuclei themselves by a path. However, RMDs do not surround the nucleus in either system [79].

## 4. Discussion

### 4.1. Characterization of GO

In the XRD analysis, the peak in GO is greater than that of GOr, due to the incorporation of oxygen functional groups and intercalated water. This result agrees with that reported in other studies [80], where the increase in interlayer spacing facilitates exfoliation and improves dispersibility in aqueous solutions, a desirable property for applications in composites and adsorption materials.

The rough and folded morphology observed in SEM analysis is related to surface defects due to the deviation from the sp^2^ to sp^3^ character as a consequence of the high density of oxygen functional groups [91]. According to the literature, the use of ultrasound during the exfoliation of graphite oxide sheets to generate layers of graphene oxide, as well as the thermal treatment used during the drying process, could cause deformations in the structure of the sheets and their folding [80]. Moreover, the elemental composition, with a C/O ratio close to 1.25, is comparable to that reported in the literature [92], reflecting significant oxidation but preserving the essential carbonaceous structure for conductivity and mechanical strength.

Regarding the FTIR analysis, it is evident that hydroxyl, epoxy, carboxyl, and carbonyl are the dominant functional groups in GO. Thus, the band at around 2900 cm^−1^ represents the C-H stretching of heterocyclic compounds. The bands observed at 1714 cm^−1^ and 1575 cm^−1^ depict the vibrations of the stretching carboxylic acid group (C=O) and C=C aromatic stretching, respectively. GO also showed strong characteristic peaks at 1377 cm^−1^, 1219 cm^−1^, and 1031 cm^−1^, corresponding to the bending vibrations of C–OH, and stretching vibrations of both C–O in C–OH and C–O–C (epoxy), respectively. The band at around 500 cm^−1^ may correspond to C–O stretching vibrations. These results agree with those reported by other authors [93]. The broad peak between 3000 and 3600 cm^−1^ is due to the vibration of O–H stretching for carboxyl and hydroxyl groups and the water molecules adsorbed between the GO sheets. From these results, it can be suggested that the most likely configuration of the graphene oxide sheet consists of a combination of clean graphene regions and fully oxidized regions where the functional groups would be locally arranged in a random manner. However, due to the highly unstable nature of the oxidation process, several intermediate phases may still exist in the experimental samples. In the same context of FTIR analysis, GO was evaluated after adsorbing MB. The results indicate that, in addition to the characteristic peaks of GO, there are new peaks that would be related to the structure of MB. The new peaks were observed at 1540 cm^−1^, 1458 cm^−1^, 1397 cm^−1^, and 1335 cm^−1^, corresponding to the C=C stretching, C=N stretching, C–H_3_ bending, and C–N stretching vibrations of the terminal saturated dimethyl amino group, respectively [94]. Furthermore, the adsorption of methylene blue (MB) on graphene oxide (GO) induces characteristic changes in the FTIR bands of GO, providing detailed information on the underlying adsorption mechanisms. The decrease in intensity in the O-H band at around 3400 cm^−1^ suggests that the hydroxyl groups of GO are involved in the formation of hydrogen bonds with MB, while the changes in the C=O stretching band at around 1720 cm^−1^ could indicate interactions between the carboxylic groups of GO and the positive charges of MB, consistent with electrostatic interactions. Furthermore, the alteration in the bands associated with C=C bonds at around 1620 cm^−1^ reflects possible π–π interactions between the aromatic rings of GO and MB [94]. These spectral changes not only confirm the adsorption of MB on the GO surface, but also highlight the synergistic role of hydrogen bonds, electrostatic forces and π–π interactions in the adsorption process, which is important for the efficient removal of organic contaminants in aqueous solutions.

In this study, the specific surface area (SSA = 178.4 m^2^ g^−1^), monolayer volume (MV = 40.9 cm^3^ g^−1^) and point of zero charge (PZC = 5.5) of the synthesized GO were also evaluated. SSA is an important indicator of the adsorbent capacity of a material, with higher values generally indicating a better adsorption performance due to the larger surface area available for interaction with the adsorbate molecules. Compared to other graphene-based materials reported in the literature, with SSA in the range of 30–880 m^2^ g^−1^ [95], the GO synthesized here shows good potential, especially considering that its surface functionalities offer specific sites for the adsorption processes. This balance between the surface area and chemical functionality of GO is crucial for applications where a specific interaction with the adsorbate is desired, such as in the selective removal of contaminants. On the other hand, the results of PZC shown in Figure S1, suggest that the surface of the adsorbent will be positively charged when the pH of the solution is lower than 5.5, and negatively charged when the pH of the solution is superior to this value. Therefore, a pH > pH_PZC_ would promote favorable electrostatic interactions between the adsorbent surface and cationic molecules, such as MB dye [96]. The combination of PZC and SSA in the characterization of GO offers a comprehensive view of how this material can be optimized for specific applications. For instance, in contaminant adsorption processes, understanding the PZC allows for pH adjustment to maximize electrostatic interactions, while a high SSA ensures the wide availability of adsorption sites. However, it is important to note that the presence of oxygen functional groups, as evidenced in the FTIR analysis, can impact both the PZC and SSA by introducing porosity and increasing the dispensability of the material, which could affect the accessibility of the pores and the charge distribution on the GO surface. Additionally, the interaction between PZC and SSA under different environmental conditions can significantly influence the stability and reusability of GO. For example, in highly acidic or basic environments, modifications to the surface charge can not only affect the adsorption efficiency but also the structural stability of the material, which is critical for repetitive adsorption applications.

### 4.2. Experimental Adsorption Studies

#### 4.2.1. Effect of pH on MB Adsorption

The effect of pH on the adsorption of MB dye on graphene oxide (GO) is a fundamental aspect of understanding the interaction between the adsorbate and the adsorbent, as well as optimizing the adsorption efficiency in practical applications. The adsorption process depends on the pH of the solution, since this influences both the distribution of surface charges on the adsorbent and the speciation of the dye in the solution [97]. According to the literature, molecular MB can exist in an aqueous solution as an undissociated molecule (MB°) and as a cationic species (MB^+^). At pH = 3, the MB° form predominates, at pH = pKa = 3.8, MB° and MB^+^ coexist in equal proportions, and at pH > 6, the cationic form MB^+^ is practically the only species present [98].

In this study, it was observed that GO exhibits a higher MB adsorption capacity as the pH of the solution increases, up to an optimum close to pH = 7.0. However, at extremely basic pH values, the adsorption capacity drastically decreases, which can be attributed to the destabilization of GO and the creation of positively charged sites on its surface, leading to electrostatic repulsion with MB molecules [99,100]. Furthermore, according to the literature, the molecular structure of MB could be modified at a high pH due to gradual demethylation [101]. The variation in the adsorption capacity of MB with pH can be explained by the modification of the surface charge of GO and the ionization of MB molecules. At a low pH, the acidic environment facilitates the protonation of oxygen functional groups on the GO surface, generating a positive surface charge that can repel cationic MB. As the pH increases, these groups are deprotonated, giving rise to a more negatively charged surface that favors the adsorption of MB through electrostatic attraction.

The high adsorption capacity observed at alkaline pH values can be attributed to the increase in hydroxyl ions, resulting in greater electrostatic attraction between the MB^+^ cationic species and the negatively charged surface of graphene oxide [102]. However, at extremely high alkaline pH levels, OH ions can form complexes with other ions, such as MB^+^, potentially influencing dye adsorption on the adsorbent surface [103]. This phenomenon could result in the precipitation of dye molecules on the GO surface. Consequently, the adsorption mechanism at an alkaline pH is likely to be a combination of electrostatic attraction and precipitation [104]. On the other hand, Figure 6 also shows that graphene oxide has a reasonably good MB+ adsorption capacity at pH < pH_PZC_, where electrostatic interactions do not favor adsorption. Under these experimental conditions (pH < 5.5), it is suggested that the adsorption of the dye could occur via ion exchange since the cationic species MB^+^ would be competing with H^+^ for the active sites on the GO surface [18].

#### 4.2.2. Effect of Initial Concentration of MB

The adsorption capacity of GO was evaluated using Langmuir, Freundlich, and Temkin adsorption isotherms, and a favorable fit was found with the Langmuir model, suggesting monolayer adsorption on homogeneous surfaces of GO. This behavior indicates a specific and uniform interaction between MB and the active sites on GO, which is crucial for purification applications where high selectivity and efficiency are required. From the Langmuir model, the maximum adsorption capacity (q_max_) was estimated, reflecting the maximum amount of MB that can be adsorbed per unit mass of GO under optimal conditions. Furthermore, in this study, it was found that the value of the Langmuir separation factor (R_L_) was in the range of 0 < R_L_ < 1 for the tested initial MB concentrations, indicating that adsorption is favorable under the experimental conditions that were used. Values of R_L_ between 0 and 1 suggest favorable adsorption, while R_L_ > 1 would indicate unfavorable adsorption [105]. The fit of the data to both the Langmuir model, and the R_L_ values indicates not only the feasibility and favorability of MB adsorption on GO but also that surface saturation occurs in a monolayer. This is indicative of a high affinity between MB and GO, possibly due to π–π interactions between the aromatic rings of MB and the graphene structure of GO, as well as possible electrostatic interactions depending on the pH of the solutions. The coefficients obtained from the Freundlich model, especially the value of n, which falls between 1 and 10, suggest that the adsorption is physical and favorable. The physical nature of adsorption is beneficial from the perspective of adsorbent reusability, as physical interactions are generally easier to reverse than chemical ones, facilitating the regeneration of GO.

#### 4.2.3. Effect of Temperature on MB Adsorption

The effect of temperature on the adsorption of methylene blue (MB) on graphene oxide (GO) is a key factor in understanding the thermodynamic nature of the adsorption process. In this study, it was observed that, with an increase in temperature, the adsorption capacity of MB on GO also increases, indicating that adsorption is an endothermic process. This behavior suggests that at higher temperatures, there is a greater affinity between MB and GO, probably due to the increased mobility of the MB molecules and the expansion of GO pores, which facilitates access to more adsorption sites. Additionally, the obtained thermodynamic parameters, such as the change in Gibbs free energy (ΔG°), enthalpy (ΔH°), and entropy (ΔS°), confirmed the endothermic nature of the process. Negative values of ΔG° at different temperatures indicate that adsorption is spontaneous, while a positive value of ΔH° reinforces the idea that the process is endothermic. The positive increase in ΔS° suggests an increase in disorder at the solid–liquid interface during adsorption, which is typical in physical adsorption processes.

#### 4.2.4. Effect of Contact Time on MB Adsorption

This study reveals that the adsorption kinetics of MB on GO follow the pseudo-second-order model, indicating that the rate of adsorption depends on the amount of MB adsorbed at equilibrium and the amount of MB adsorbed at any given time. According to the pseudo-second-order model, rate of MB adsorption increases over time until equilibrium is reached, which fits well with the model, suggesting that the rate-limiting step is the chemical interaction between MB and the active sites on the GO surface. The high correlation coefficient R^2^ values for this model indicate a good fit of the experimental data, reflecting the applicability of the model in describing the adsorption kinetics of the GO-MB system. The fit with the pseudo-second-order model underscores the characteristics of GO as a material with active sites capable of chemically interacting with MB molecules, facilitating efficient adsorption. This observation is in line with the implications of the Langmuir isotherm observed in this study, where the formation of a monolayer of MB on the GO surface indicates that adsorption occurs at specific sites with a uniform adsorption energy.

On the other hand, MB adsorption on GO showed typical intraparticle diffusion behavior, where three distinct stages were identified based on the kinetic constant of each stage. The first stage, characterized by a high kinetic constant, corresponds to the external diffusion of MB towards the GO surface. The second stage, with a lower kinetic constant, reflects the intraparticle diffusion of MB within the pores of GO. Finally, the last stage, with the lowest kinetic constant, indicates adsorption equilibrium, where the rate of adsorption slows down due to the saturation of adsorption sites. The diffusion coefficients in the external film phase (D_f_) and the adsorbent phase (D_p_) provide information on the resistance to mass transfer during the adsorption process. In this study, the values of D_f_ and D_p_ indicate that intraparticle diffusion and mass transfer through the external film boundary significantly contribute to the adsorption kinetics, which is consistent with the results obtained in the kinetic modeling. The relationship between these coefficients and the results reflects the complexity of the adsorption process, where both diffusion mechanisms (intraparticle and through the film boundary) play important roles in the overall rate of adsorption. This highlights the need to optimize operational conditions, such as agitation and adsorbent particle size, to minimize resistance to mass transfer and maximize adsorption efficiency.

Finally, evidence from the experimental study showed that the maximum MB adsorption capacity (q_max_) of the synthesized GO is comparable to that of other adsorbents, as previously reported by other authors (Table 7).

A comparison of the methylene blue (MB) adsorption capacity of graphene oxide (GO) synthesized in this study, with a capacity of 163 mg g^−1^, against other adsorbents reported in the literature, offers a detailed perspective on the performance of different materials in the adsorption of contaminants. The GO in this study shows an intermediate performance compared to other forms of GO, with capacities ranging from 145 mg g^−1^ to 397 mg g^−1^ for the 3D GO sponge, suggesting that specific structural modifications, such as increasing the three-dimensionality, can significantly improve the adsorption capacity. This is probably due to an increase in the surface area available for adsorption and possibly the presence of more active sites. On the other hand, reduced graphene oxide (rGO) shows wide variability in its capacity, from as low as 68 mg g^−1^, indicating how reduction treatments significantly affect its adsorption properties, possibly altering the quantity and nature of the functional groups on the surface of the material. Compared with other types of activated carbon and nanomaterials, such as coconut-shell-activated carbon with 200 mg g^−1^ and multi-walled carbon nanotubes with 48 mg g^−1^, it is evident that the GO synthesized here shows a competitive ability, especially considering that nanotubes and activated carbon are highly optimized for adsorption. Differences in the adsorption capacity of the tested materials can be attributed to variations in their morphology, surface area, porosity, and surface chemistry. Therefore, to improve the adsorbent capacity of synthesized GO in future research, it would be beneficial to explore structural modifications, such as creating nanoscale pores or increasing porosity through chemical or physical activation techniques. Furthermore, functionalization of the GO surface with chemical groups that have a high affinity for MB could increase the specific adsorption capacity of this compound. It would also be interesting to investigate the synergy between GO and other materials in hybrid composites, which could combine the unique properties of various adsorbents to achieve superior adsorption capacities and specific selectivity for different contaminants.

### 4.3. Computational Studies

#### 4.3.1. Optimization of Adsorbent Structures

This study focused on analyzing how the introduction of hydroxyl and epoxy functional groups affects the structural and electronic properties of GO, and therefore, its adsorption capacity. The findings reveal that the functionalization of GO with these groups can significantly modify both the morphology and surface chemistry of the material, which has direct implications for its performance as an adsorbent.

The introduction of hydroxyl and epoxy groups into the structure of GO was optimized to increase the efficacy in the adsorption of MB molecules. These functional groups not only improve the dispersibility of GO in water but also offer specific sites for adsorption through hydrogen bonding and van der Waals forces interactions. The optimized structure of GO showed a significant improvement in adsorption capacity compared to non-functionalized graphene, which is attributed to the increased available surface area and the presence of active sites for adsorption.

#### 4.3.2. Electronic Properties of Adsorbent Structures

The introduction of oxygen functional groups into graphene, such as hydroxyls and epoxys, induces significant changes in the electronic structure of the material, which are reflected in the density of states (DOS) and the band structure (BS). These changes have profound implications for the hybridization of orbitals, the Fermi level, and the Dirac point, key elements for understanding and applying the material in electronic devices and sensors. Pure graphene possesses an sp^2^ hybridization of carbon orbitals, which gives it a flat structure and unique electronic properties, such as high electrical conductivity. The introduction of oxygen functional groups disturbs this sp^2^ hybridization, introducing regions of sp^3^ hybridization. This results in a local distortion of the graphene flat structure and the formation of defective electronic states that affect the mobility of charge carriers and the optical and electrical properties of the material. The Fermi level in pure graphene is located at the Dirac point, where the conduction and valence bands meet, resulting in a null bandgap and high intrinsic conductivity.

The functionalization of graphene with oxygenated groups can shift the Fermi level due to charge transfer between graphene and the functional groups. This shift modifies the conduction properties of the materials, as the Fermi level dictates the occupancy of available electronic states and, therefore, the conductivity. The Dirac point, characteristic of graphene, is the crossing point of the conduction and valence bands in the band diagram, which are crucial for the unique electronic properties of graphene. In this study, the functionalization of graphene with oxygenated groups produced a slight perturbation at the Dirac point, as electronic states were introduced into the bandgap, potentially opening a small energy gap at the Dirac point. This change could certainly reduce the electronic mobility of the material but increases its semiconductivity, allowing for graphene to be adapted for applications that require a bandgap, such as in transistors or gas sensors.

Several authors have shown how the introduction of defects and functional groups alters the electronic properties of graphene and improves its adsorption capacity, corroborating the findings of this study. This phenomenon is probably due to the introduced functional groups, which could increase the chemical compatibility between the adsorbent and adsorbate, facilitating the adsorption process [118,119]. Therefore, the results of this study support the idea that the functionalization of graphene is an effective strategy to increase its versatility and effectiveness as an adsorbent material.

#### 4.3.3. Computational Adsorption Studies

The computational study of the molecular adsorption of methylene blue (MB) on graphene (G) and graphene oxides (GO) sheds light on how these interactions can be optimized for applications such as water purification and sensor design. Table 7 provides detailed data on the adsorption of MB on graphene and graphene oxide (GO) surfaces, highlighting differences in adsorption energy, interfacial distance, and charge transfer. These parameters are crucial for understanding the interaction between MB and the different graphene-based materials. The adsorption energy is an indicator of the strength of the interaction between the adsorbate (MB) and the adsorbent (G or GO). The negative values achieved in this study indicate that all adsorptions are spontaneous processes. The adsorption energy of −25.96 kJ/mol, calculated for the G-MB system, indicates a moderately strong interaction, suggestive of predominant physical interactions, such as van der Waals forces and π–π stacking between the aromatic rings of MB and the graphene surface. In the GOL-MB system, the adsorption energy significantly increases to −67.27 kJ/mol, indicating a much stronger interaction compared to pure graphene. This suggests that the presence of hydroxyl groups on GO facilitates additional interactions, possibly through hydrogen bonding, increasing the adsorbent affinity for MB. Finally, the adsorption energy of −53.53 kJ/mol for the GOXI-MB system also shows a strong interaction, although slightly less than that of GO with hydroxyl groups. This could be due to the specific characteristics of the epoxy groups, which also allow for strong interactions, but with a different bonding dynamic compared to hydroxyls.

The interfacial distance between MB and the adsorbent surface provides an idea of how close the molecules are to each other, which directly affects the nature of the interaction. The interfacial distance of 3.05 Å in the G-MB system is consistent with non-covalent interactions, such as van der Waals forces and π–π stacking. In contrast, the shorter distances of 2.47 Å and 2.26 Å calculated for the GOL-MB and GOXI-MB systems, respectively, suggest stronger interactions, likely due to the formation of hydrogen bonds between the functional groups of GO and MB, in addition to π–π interactions.

The charge transfer from the adsorbent to the adsorbate (or vice versa) is an indicator of the formation of a chemical bond or significant electrostatic interactions. The charge transfer of ±0.39 e in the G-MB system indicates a moderate interaction, consistent with van der Waals forces and π–π stacking. Meanwhile, the more significant charge transfers of ±0.68 e and ±0.57 e from the GOL-MB and GOXI-MB systems, respectively, suggest a stronger chemical interaction, possibly due to the formation of hydrogen bonds and increased polarization at the interface.

It is important to mention that the horizontal orientation of the MB molecule on the G and GO surfaces adopted in this study is justified by maximizing the π–π interactions between the aromatic systems of MB and the graphene-based structures. This adsorption mechanism is consistent with the findings of other studies, which highlight how the horizontal orientation facilitates a larger contact surface, increasing the interaction between the adsorbate and the adsorbent [120,121]. The choice of this orientation is supported by molecular dynamics simulations and total energy calculations that show an energetic preference for the horizontal arrangement over vertical or inclined configurations. The ELF analysis reinforces this interpretation, showing a redistribution of electronic density that favors the formation of weak bonds and potentially hydrogen bonds between MB and the functional groups of GO. The ELF analysis provides a detailed view of the electronic distribution at the adsorbate–adsorbent interface, revealing areas of high electronic density indicative of possible chemical interaction sites. In the case of the horizontal orientation of MB, the ELF analysis suggests that π–π interactions between the aromatic rings of MB and graphene/GO are prominent, with the possible participation of hydrogen bonds between the oxygenated groups of GO and the methyl groups of MB. This type of interaction contributes to a more stable and stronger adsorption, which is crucial for adsorption efficiency.

The experimental and theoretical results of this study demonstrate that the functionalization of graphene with hydroxyl and epoxy groups significantly increases its efficiency as an adsorbent material, not only by improving the existing physical interactions (π–π stacking), but also by introducing new chemical interactions (hydrogen bonds). From the experimental results, it can be inferred that the modified GO exhibits optimal adsorption capacity under alkaline conditions and at elevated temperatures, suggesting that adsorption is an endothermic process and that the interactions between GO and MB are strengthened with increasing temperature. The influence of pH can be attributed to changes in the surface charge of GO and the ionization of MB, favoring adsorption through electrostatic interactions in alkaline environments. Furthermore, it was observed that the adsorption capacity increases with the initial concentration of MB until reaching equilibrium, indicating the saturation of the available adsorption sites in the GO. The contact time also plays a crucial role, as the amount of adsorbed MB was found to increase with time until equilibrium is reached, reflecting the dynamics of the interaction between the adsorbate and the adsorbent. From the theoretical perspective, the use of density functional theory (DFT) allowed for GO-MB interactions to be explored at the atomic and molecular levels, providing a deep understanding of the underlying mechanisms. DFT calculations revealed that oxygen functional groups on GO facilitate the more effective adsorption of MB by creating specific adsorption sites and enhancing electrostatic and hydrogen bonding interactions. A simulation of the modified GO structures showed how surface modifications alter the electronic properties of the material, directly influencing its interaction with MB. These theoretical results not only corroborate the experimental findings but also provide a framework to understand how the structural and functional characteristics of GO contribute to its effectiveness as an adsorbent material.

**Figure 17:**
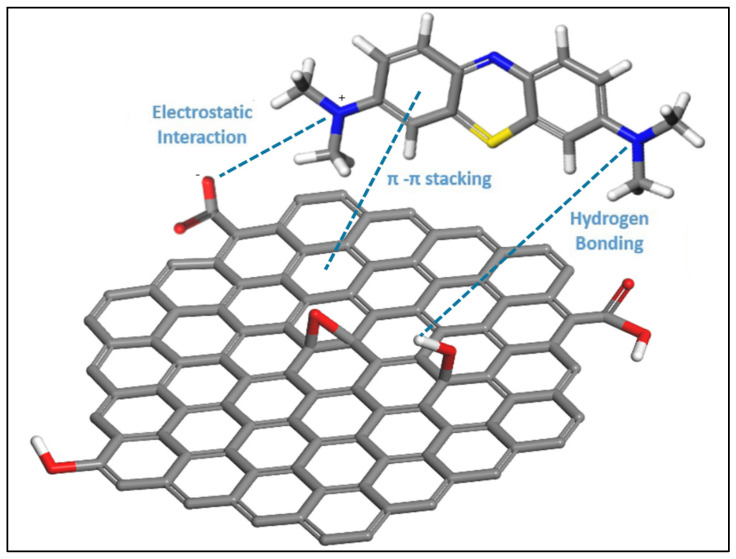
Proposed mechanism for MB adsorption on GO. The gray sticks are C bonds, the red sticks are O bonds, the white sticks are H bonds, the blue sticks are N bonds, and the yellow sticks are S bonds.

Figure 17 illustrates the proposed mechanism for the adsorption of methylene blue on graphene oxide. The figure represents the three key interactions: electrostatic interactions, hydrogen bond formation, and π–π interactions.

## 5. Conclusions

This study furthers the understanding of the adsorption capabilities of modified graphene oxide (GO) for the removal of methylene blue (MB) dye from aqueous solutions, focusing on the implications for water purification and environmental remediation. The successful modification of GO via the incorporation of oxygen functional groups, such as hydroxyls and epoxides, transforms both the structure and surface chemistry of the material. This transformation, evidenced through characterization techniques such as XRD and SEM/EDS/FTIR, results in an increase in the interlaminar distance and the introduction of surface defects, which are crucial to improve the adsorption capacity of GO.

The research reveals that the underlying adsorption mechanisms benefit significantly from the presence of these functional groups, which not only increase the hydrophilicity of GO, facilitating its dispersion in aqueous media, but also provide specific sites for the adsorption of MB molecules through hydrogen bonds and electrostatic interactions. Furthermore, the influence of factors such as pH, temperature, the initial concentration of MB, and contact time on the adsorption efficiency was highlighted, showing that alkaline conditions and elevated temperatures favor the interaction between GO and MB, highlighting the endothermic character of the adsorption process.

From a theoretical perspective, density functional theory (DFT) calculations provide a detailed understanding of the molecular interactions between GO and MB, corroborating and complementing the experimental findings. These calculations suggest that surface modifications to GO not only favor the creation of specific adsorption sites but also alter the electronic properties of the material, thus improving its interaction with MB. This detailed understanding of GO-MB interactions at the molecular level lays the foundation for the rational design of carbon-based materials with optimized adsorption properties for specific applications in water treatment and environmental remediation.

Finally, this study demonstrates the potential of modified GO as an efficient adsorbent material for the removal of organic contaminants from aqueous solutions, marking a significant advance in the search for sustainable and effective solutions to the global challenges of water purification and environmental remediation. The findings underline the importance of careful chemical modification and the optimization of operating conditions to maximize adsorption efficiency, opening new avenues for the future development of more advanced water treatment technologies.

## Figures and Tables

**Table 1:** Summary of the mathematical equations used in this study for data analysis.

Denomination	Equation		Parameters
Adsorbate adsorbed	$q_{e}=\left(C_{0}-{\;C}_{e}\right)\times \frac{v}{w}$	(1)	C_0_ = Initial concentration (mg L^−1^) Ce = Equilibrium concentration (mg L^−1^) w = Mass of the adsorbent (g) v = Volume of the solution (L)
Langmuir	$\frac{C_{e}}{q_{e}}=\frac{1}{K_{L}q_{\max}}+\frac{C_{e}}{q_{\max}}$	(2)	q_max_ = Maximum monolayer adsorption (mg g^−1^) K_L_ = Equilibrium Langmuir constant related to the adsorption energy (L mg^−1^) C_e_ = Concentration of adsorbate in solution at equilibrium (mg L^−1^)
Freundlich	qe= KFCe1n	(3)	K_F_ = Freundlich constant (L mg^−1^) 1/n = Adsorption intensity constant. Note: For favorable adsorption, the value of n should be between 1 and 10
Temkin	$q_{e}=\;B\;\ln\left({\mathrm{AC}}_{e}\right)$	(4)	q_e_ = Adsorbate adsorbed per unit weight (mg g^−1^) at equilibrium A = Temkin isotherm constant (L g^−1^) C_e_ = Concentration of adsorbate in solution at equilibrium (mg L^−1^) B = Constant related to the heat adsorption
Constant of heat adsorption	$B=\frac{\mathrm{RT}}{b}$	(5)	b = Temkin constant (J mol^−1^) T = Absolute temperature (K) R = Gas constant (8.314 J mol^−1^ K^−1^)
Separation factor	$R_{L}=\frac{1}{\left(1+K_{L}C_{e}\right)}$	(6)	K_L_ = Equilibrium Langmuir constant related to the adsorption energy (L mg^−1^) C_e_ = Concentration of adsorbate in solution at equilibrium (mg L^−1^) Note: 0 < R_L_ < 1, suitable adsorption, R_L_ > 1 suitable adsorption, R_L_ = 0 irreversible adsorption, R_L_ = 1 linear adsorption.
Gibbs free energy	$\Delta G^{0}=-{\mathrm{RT}\;\ln\;k}_{C}$	(7)	∆G^0^ = Gibbs free energy (kJ mol^−1^), ∆H^0^ = Enthalpy (kJ mol^−1^) ∆S^0^ = Entropy (kJ mol^−1^ K^−1^)
Van’t Hoff equation	$\ln k_{C}=\frac{-\Delta H^{0}}{R}\times \frac{1}{T}+\frac{\Delta S^{0}}{R}$	(8)	k_C_ = Dimensionless parameter T = Absolute temperature (K) R = Universal gas constant (8.314 J mol^−1^ K^−1^)
	$k_{C}={\;k}_{L}\times M_{w}\times 1000\times 55.5$	(9)	k_L_ = Langmuir constant (L mg^−1^) M_w_ = Adsorbate weight (g mol^−1^)
Pseudo first order	$\ln\left(q_{e}-q_{t}\right)=\ln\left(q_{e}\right)-{\;k}_{1}t$	(10)	k_1_ = Rate constant (min^−1^) q_e_ = Adsorbate adsorbed per unit weight (mg g^−1^) at equilibrium q_t_ = Adsorbate adsorbed per unit weight (mg g^−1^) at any time (t)
Pseudo second order	$\frac{t}{q_{t}}=\frac{1}{k_{2}q_{e}^{2}}+\frac{1}{q_{e}}t$	(11)	k_2_ = Rate constant (g mg^−1^ min^−1^) q_e_ = Adsorbate adsorbed per unit weight (mg g^−1^) at equilibrium q_t_ = Adsorbate adsorbed per unit weight (mg g^−1^) at any time (t)
Elovich	$q_{t}=\frac{1}{\beta }\ln\left(\alpha \beta \right)+\frac{1}{\beta }\ln\left(t\right)$	(12)	q_t_ = Adsorbate adsorbed per unit weight (mg g^−1^) at any time (t) α = Constant related to chemisorption rate β = Constant which depicts the extent of surface coverage
Intraparticle-diffusion	qt= k3t12+A	(13)	k_3_ = Intraparticle diffusion rate constant (mg g^−1^ min^−1/2^) A = constant indicating the width of the boundary layer (mg g^−1^). The larger the value of A, the greater the boundary layer effect.
Particle-diffusion	$-\ln\left(1-{\left(\frac{q_{t}}{q_{e}}\right)}^{2}\right)=\frac{2\pi^{2}D_{p}}{r^{2}}\;t$	(14)	q_e_ = Adsorbate adsorbed per unit weight (mg g^−1^) at equilibrium q_t_ = Adsorbate adsorbed per unit weight (mg g^−1^) at any time (t) *C*_z_ = Ion concentration o the adsorbent (mg kg^−1^). D_p_ = Diffusion coefficient in the adsorbent phase (m^2^ min^−1^) r = Average radius of the adsorbent particles (1 × 10^−7^ m) t = Contact time (min)
External-film-diffusion	$-\ln\left(1-\left(\frac{q_{t}}{q_{e}}\right)\right)=\frac{D_{f}C_{s}}{{h\;r\;C}_{z}}\;t$	(15)	q_e_ = Adsorbate adsorbed per unit weight (mg g^−1^) at equilibrium q_t_ = Adsorbate adsorbed per unit weight (mg g^−1^) at any time (t) D_f_ = Diffusion in the film phase surrounding the adsorbent particles (m^2^ min^−1^) *C*_s_ = Ion concentration in the solution (mg L^−1^) h = Film thickness around the adsorbent particles (10^−6^ m in poorly stirred solutions) r = Average radius of the adsorbent particles (1 × 10^−7^ m) t = Contact time (min)
Adsorption energy	$E_{\mathrm{ads}}=E_{\mathrm{sorb}/\mathrm{surf}}-E_{\mathrm{surf}}-E_{\mathrm{sorb}}$	(16)	E_sorb/surf_ = Energy of the supersystem produced by the adsorbed molecule on the surface (eV) E_surf_ = Energy of the surface (eV) E_sorb_ = Energy of the isolated molecule in vacuum (eV)

**Table 2:** Isotherm parameters for MB adsorption on GO at different temperatures.

Isotherm Parameters		293.15 K	303.15 K	313.15 K
Langmuir	q_max_ (mg g^−1^)	163.30 (±5.90)	197.94 (±6.56)	214.27 (±6.95)
	K_L_ (L mg^−1^)	0.13 (±0.02)	0.17 (±0.03)	0.21 (±0.03)
	R_L_	0.28	0.23	0.19
	χ^2^	6.05	6.33	6.79
	R^2^	0.98	0.97	1.00
Freundlich	K_F_ (L mg^−1^)	34.09 (±2.98)	38.22 (±2.94)	44.72 (±2.74)
	N	2.59 (±0.45)	2.90 (±0.49)	3.40 (±0.53)
	1/n	0.39	0.34	0.29
	χ^2^	2.42	3.12	3.45
	R^2^	0.90	0.92	0.95
Temkin	B	34.39 (±2.87)	38.55 (±2.24)	45.12 (±2.98)
	A	1.39 (±0.31)	1.55 (±0.42)	1.82 (±0.45)
	χ^2^	2.92	3.06	3.86
	R^2^	0.96	0.93	0.96

**Table 3:** Thermodynamic parameters of the MB adsorption onto GO.

Temperature (K)	ln k_C_	∆G° (kJ mol^−1^)	∆H° (kJ mol^−1^)	∆S° (kJ mol^−1^ K^−1^)
293.15	12.18	−29.69	18.21	0.16
303.15	12.40	−31.25		
313.15	12.66	−32.96		

**Table 4:** Kinetic parameters for MB adsorption onto GO.

Kinetic Parameters		293.15 K
Pseudo-first-order	q_max_ (mg g^−1^)	292.39 (±5.57)
	k_1_ (L mg^−1^)	0.02 (±1.77 × 10^−3^)
	χ^2^	6.84
	R^2^	0.97
Pseudo-second-order	q_max_ (mg g^−1^)	332.29 (±2.30)
	k_2_ (L mg^−1^)	9.01 × 10^−5^ (±3.33 × 10^−6^)
	χ^2^	11.21
	R^2^	1.00
Elovich	A	30.82 (±5.61)
	Β	0.02 (±9.39 × 10^−4^)
	χ^2^	6.34
	R^2^	0.98
Intraparticle diffusion	k (mg g^−1^ min^−1/2^)	145.18 (±8.54)
	A	44.53 (±7.12)
	R^2^	0.91
External-film diffusion	D*f* (m^2^ min^−1^)	6.12 × 10^−12^
	R^2^	0.89
Internal-pore diffusion	D*p* (m^2^ min^−1^)	1.44 × 10^−18^
	R^2^	0.91

**Table 5:** Calculated values of absorption energy, interfacial distance, and transferred charge.

Optimized System	Adsorption Energy (kJ mol^−1^)	Interfacial Distance (Å)	Charge Transfer (e)	
			Surface	MB
G-MB	−25.96	C-H_MB_ = 3.05	+0.39	−0.39
GOL-MB	−67.27	H-S_MB_ = 2.47	+0.68	−0.68
GOXI-MB	−53.53	O-H_MB_ = 2.26	+0.57	−0.57

**Table 6:** Calculated values of transferred charge.

Atoms	G-MB			GOL-MB			GOXI-MB		
	BA (e)	AA (e)	ΔCharge	BA (e)	AA (e)	Δcharge	BA (e)	AA (e)	ΔCharge
C_surf_	0.0000	−0.0012	0.0012	0.0060	0.0040	0.0020	0.0141	0.0099	0.0042
O_surf_	-	-	-	−1.4837	−1.4913	0.0076	−1.1389	−0.9428	−0.1961
H_surf_	-	-	-	0.9999	0.9999	0.0000	-	-	-
C_MB_	0.0802	0.4812	−0.4009	0.0802	0.4609	−6.0916	0.0802	0.4670	−0.3868
H_MB_	0.2756	0.0186	0.2498	0.2756	0.0463	4.1264	0.4870	0.2005	0.2865
N_MB_	−2.2436	−2.6266	0.3830	−2.2436	−2.5924	0.2421	−2.2436	−2.6007	0.3395
S_MB_	0.4870	0.2372	0.2570	0.4870	0.2449	1.0465	0.2756	0.0386	0.2370

**Table 7:** Comparison of adsorption capacity with other studies.

Adsorbent	Adsorption Capacity (mg g^−1^)	Reference
Graphene Oxide	332	[106]
Graphene Oxide	287	[107]
Graphene Oxide	244	[108]
3D Graphene Oxide sponge	397	[109]
Graphite Oxide	351	[110]
Graphene	154	[111]
Reduced Graphene Oxide	68	[112]
Coconut-Shell-Activated Carbon	200	[113]
Multi-Wall Carbon Nanotube	48	[114]
Graphene Oxide	145	[115]
Magnetic Graphene Oxide	25	[116]
Fe_3_O_4_/Chitosan/Graphene	48	[117]
Graphene Oxide	163	This study

## Data Availability

Data are available from the authors upon reasonable request.

## Supplementary Materials

The following supporting information can be downloaded at: https://www.mdpi.com/article/10.3390/nano14080714/s1, Figure S1. Point of zero charge (PZC) of GO.
