# Supplementary material for: Theoretical and Experimental Analysis of Hydroxyl and Epoxy Group Effects on Graphene Oxide Properties
Source: Nanomaterials (Basel). 2024 Apr 19;14(8):714. doi: 10.3390/nano14080714 (PMC11054681; doi:10.3390/nano14080714)
Supplement: Supplementary file 1 [file nanomaterials-14-00714-s001.zip › nanomaterials-2959231-supplementary.pdf]

nanomaterials

Supplementary Materials

Theoretical and Experimental Analysis of Hydroxyl and Epoxy Group Effects on Graphene Oxide Properties

Ximena Jaramillo-Fierro [1,*] and Guisella Cuenca [2]

[1] Departamento de Química, Facultad de Ciencias Exactas y Naturales, Universidad Técnica Particular de Loja, San Cayetano Alto, Loja 1101608, Ecuador

[2] Ingeniería Química, Facultad de Ciencias Exactas y Naturales, Universidad Técnica Particular de Loja, San Cayetano Alto, Loja 1101608, Ecuador; gpcuenca@utpl.edu.ec

* Correspondence: xvjaramillo@utpl.edu.ec; Tel.: +593-7-3701444

Abstract: In this study, we analyzed the impact of hydroxyl and epoxy groups on the properties of graphene oxide (GO) for the adsorption of methylene blue (MB) dye from water, addressing the urgent need for effective water purification methods due to industrial pollution. Employing a dual approach, we integrated experimental techniques with theoretical modeling via density functional theory (DFT) to examine the atomic structure of GO and its adsorption capabilities. The methodology encompasses a series of experiments to evaluate the performance of GO in MB dye adsorption under different conditions, including pH, dye concentration, reaction temperature, and contact time, providing a comprehensive view of its effectiveness. Theoretical DFT calculations provide insights into how hydroxyl and epoxy modifications alter the electronic properties of GO, improving adsorption efficiency. The results demonstrate a significant improvement in the dye adsorption capacity of GO, attributed to the interaction between the functional groups and MB molecules. This study not only confirms the potential of GO as a superior adsorbent for water treatment, but also contributes to the optimization of GO-based materials for environmental remediation, highlighting the synergy between experimental observations and theoretical predictions in advancing the sustainability of materials science.

Keywords: adsorbent materials; graphene oxide; methylene blue; density functional theory; water purification; environmental remediation

Citation: Jaramillo-Fierro, X.; Cuenca, G. Theoretical and Experimental Analysis of Hydroxyl and Epoxy Group Effects on Graphene Oxide Properties. *Nanomaterials* **2024**, *14*, 714. https://doi.org/10.3390/nano14080714

Academic Editor: Thomas Dippong

Received: 26 March 2024
Revised: 13 April 2024
Accepted: 16 April 2024
Published: 19 April 2024

Copyright: © 2024 by the authors. Submitted for possible open access publication under the terms and conditions of the Creative Commons Attribution (CC BY) license (https://creativecommons.org/licenses/by/4.0/).

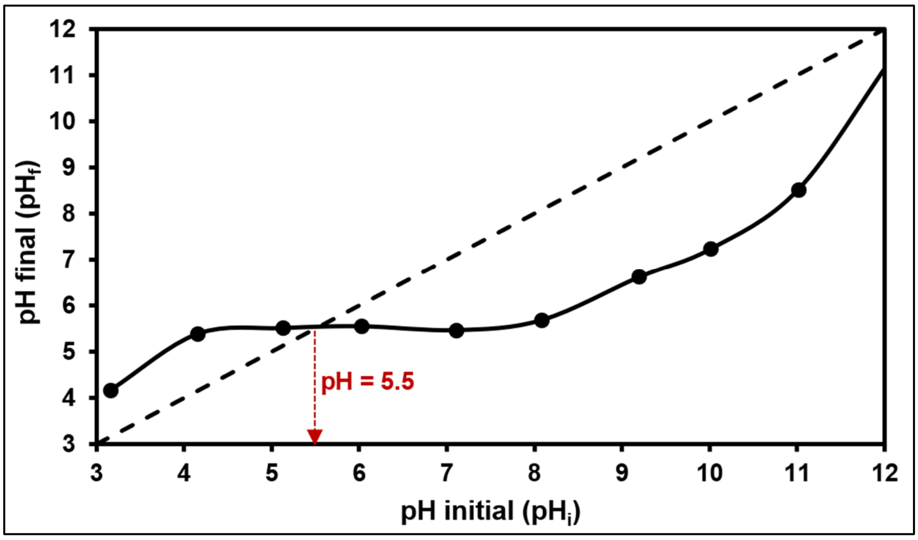

Figure S1. Point of zero charge (PZC) of GO.

Disclaimer/Publisher's Note: The statements, opinions and data contained in all publications are solely those of the individual author(s) and contributor(s) and not of MDPI and/or the editor(s). MDPI and/or the editor(s) disclaim responsibility for any injury to people or property resulting from any ideas, methods, instructions or products referred to in the content.
